# Disaster mechanisms and hazard assessment of rapid and steep-gully debris flows based on FLO-2D simulation

**DOI:** 10.1371/journal.pone.0356759

**Published:** 2026-08-26

**Authors:** Haiwei Du, Runsen Lai, Jianhua Zhu

**Affiliations:** 1 School of Science and Technology, Hong Kong Metropolitan University, Hong Kong, China; 2 School of Geology and Mining Engineering, Xinjiang University, Urumqi, China; 3 Xinjiang Geological Environment Monitoring Research institute, Urumqi, China; Yogi Vemana University, INDIA

## Abstract

The kinematic evolution and precise hazard zonation of rapid debris flows in steep gullies remain challenging due to complex topographic and hydrological conditions. Taking a typical catchment in the North Tianshan Mountains as a case study, this research investigates the dynamic disaster-triggering mechanisms and spatial hazard distribution of such events by integrating field surveys, laboratory testing, and FLO-2D hydrodynamic modeling. Results indicate that formation is primarily governed by the regional geological setting and rainfall intensity. Highly fractured rock masses provide abundant source material, while high-gradient topography facilitates rapid initiation and transport. Short-term intense rainfall acts as the decisive trigger, with the kinematic evolution characterized as a “source enrichment–dynamic triggering–path conduction–accumulation” disaster chain arising from multi-factor coupling. Quantitative reconstruction of the 2024 event demonstrates that the model achieves verification accuracies of 89.01% for maximum flow depth and 87.84% for deposition area, confirming its reliability for this specific gully type. Multi-scenario hazard assessments reveal that flow depth, velocity, and hazard footprints expand significantly with increasing rainfall return periods. Under a 100-year scenario, the maximum flow depth reaches 5.83 m, the peak velocity is 6.80 m/s, and the high-hazard zone covers 2.28 × 10⁴ m², posing severe threats to downstream settlements. By providing a validated, high-precision hydrodynamic framework, this study offers a robust scientific basis for multi-scenario hazard zonation and the design of engineering mitigation strategies in vulnerable mountainous terrains.

## 1. Introduction

Debris flows are common and highly destructive geological hazards in mountainous regions, typically triggered by hydrological inputs such as rainstorms, glacial meltwater, or snowmelt. Originating in valleys or on steep slopes, these events manifest as specialized sediment-laden torrents transporting massive volumes of solid material, including silt, stones, and boulders [1,2]. Their occurrence is characterized by extreme suddenness and high velocity [3], capable of mobilizing tens of thousands—or even millions—of cubic meters of material within a remarkably short duration. Statistical data indicate that hundreds to over a thousand debris flow disasters occur annually in China, posing a severe threat to public safety and infrastructure [4]. Among these, a specific category known as “rapid and steep-gully debris flow” is particularly hazardous. These gullies are primarily located in piedmont areas with multiple nearly parallel channels. Their exceptionally steep slopes and complex topographic conditions facilitate rapid runoff convergence and the accumulation of immense potential energy [5,6]. Due to their catastrophic destructive capacity, research into the disaster mechanisms and hazard assessment of such debris flows is both urgent and essential [7–9].

In recent years, scholars have focused extensively on the genesis mechanisms and formation processes of debris flows under diverse topographic, hydrological, and meteorological conditions. Regarding disaster patterns, numerous case studies [10–16] have elucidated initiation mechanisms across various geological settings and human-induced disturbances. These studies systematically map the entire evolutionary process—from source mobilization and transport to accumulation—providing theoretical support for early warning and engineering mitigation. For hazard assessment, various evaluation models integrated with GIS technologies have enabled regional comprehensive assessments [17–23]. However, most existing research centers on “general” debris flows, while studies on the specialized “rapid and steep-gully” type remain relatively scarce. Unique topographic conditions in these gullies trigger high-speed, large-scale disasters in much shorter timeframes, making their disaster-causing processes significantly different from those of general debris flows.

As one of the three fundamental conditions for debris flow occurrence, topography exerts a decisive influence on hazard dynamics. In steep gullies, the “fire hose effect” frequently occurs during the mobilization of source materials [24–27]. This effect refers to a mechanism where high-velocity concentrated flow, formed by upstream confluence after intense rainfall, acts like a pressurized water jet to scour loose deposits with powerful inertial impact, triggering rapid initiation. These events are characterized by short lead times, high flow velocities, long runout distances, and severe impact [28]. With the increasing frequency of short-duration, high-intensity rainfall events, the occurrence of steep-gully debris flows has become more prevalent. These drainage basins are typically small, with longitudinal slope gradients often exceeding 250‰ [29,30].

Current research on steep-gully events primarily combines disaster pattern synthesis with verification via numerical simulation. Li et al. [31] investigated the development characteristics of narrow and steep-gully debris flows in the Wenchuan earthquake zone, revealing distinct topographic features and grain-size distributions. Han et al. [32] defined their unique formation mechanisms through comparative analysis with wide-and-gentle gullies. Utilizing FLO-2D, Fang et al. [8] and Chen et al. [33] demonstrated the model’s high validity and practicality for simulating along-channel dynamics in steep terrains. Other researchers have employed OPENLISM [34] and Arc-SCS [35] to predict scouring ranges and hazard footprints in high-gradient environments. Nevertheless, the applicability of existing models under extreme climatic conditions requires further verification, and research on the response of these systems to climate change remains insufficient.

**Fig 1:**
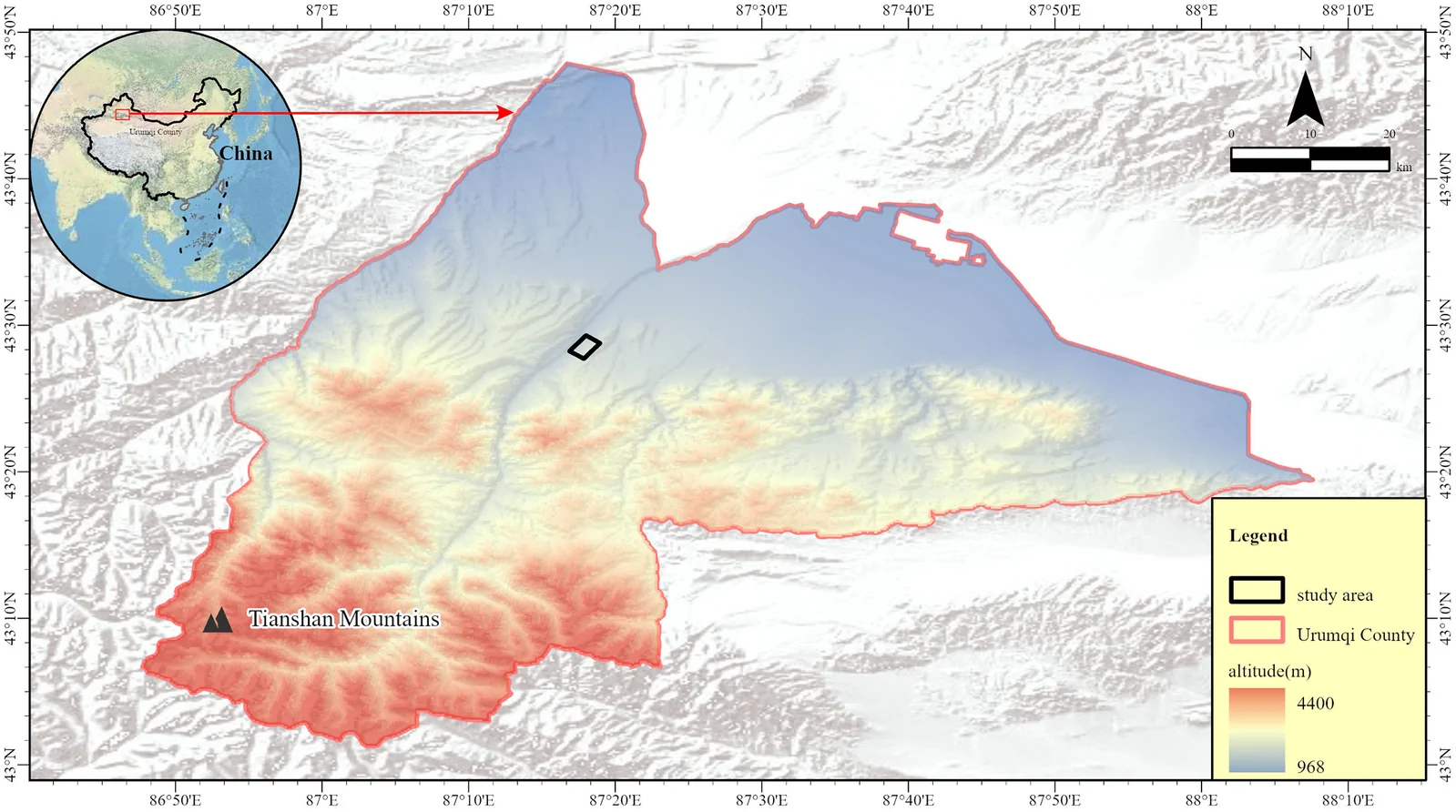
Schematic map of the study area location.

To address these gaps, this paper investigates a typical steep-gully debris flow basin on the northern slope of the Tianshan Mountains: the Dongbaiyanggou debris flow in Urumqi, Xinjiang (Fig 1). This region is a focal point for geological disaster prevention in China. By integrating field investigations(No specific permits were required as the site is public land and did not involve protected species), laboratory testing, and FLO-2D numerical simulations, this study aims to characterize the geological environment, material source distribution, and disaster patterns. The entire process is reconstructed to summarize the triggering mechanisms and quantitatively evaluate hazard under various probability scenarios. These findings provide a scientific basis for regional disaster mitigation and a reference for debris flow research in similar geological environments.

The administrative boundaries are derived from GADM (https://gadm.org/), and the macro-topographic background is generated from the ALOS PALSAR DEM (https://search.asf.alaska.edu/).

## 2. Geological setting and debris flow characteristics

### 2.1. Regional geological setting

The study area is situated on the northern slope of the Tianshan Mountains within the Urumqi River system. The terrain exhibits a distinctive strip-like distribution, transitioning from high altitudes in the south (1930 m) to lower elevations in the north (1560 m). This region is characterized by deeply incised mid-low mountain landforms with an average longitudinal profile gradient of 255‰. The flanking mountain slopes are notably steep, with gradients consistently ranging between 30° and 50°.

The regional climate is a typical continental mountain climate with a multi-year average temperature of 8.8°C. Thermal peaks occur from June to August, with average temperatures reaching 25.3°C in July. Precipitation patterns show significant interannual variability, with a mean annual value of 261.13 mm (ranging from 131.3 mm to 419.5 mm). Critically, summer rainfall accounts for 32.6%–73.3% of the annual total and predominantly occurs as high-intensity rainstorms, which facilitate rapid runoff concentration and surge flood discharge in the upper catchments.

Tectonically, although the area is distant from major fault zones, its proximity to seismically active regions results in frequent earthquakes. These seismic events have complicated the geomorphology, fostering the development of deeply incised valleys, steep terrains, and bedrock terraces—all of which provide favorable boundary conditions for debris flow initiation. The lithology comprises Permian conglomerate-dominated sedimentary rocks, alongside Quaternary Holocene alluvial-proluvial and slope deposits. The conglomerates are severely weathered, while the surface is mantled by silty and brecciated soils. These extensive soft-rock formations and loose deposits serve as the primary material sources for debris flow activity.

### 2.2. Debris flow characteristics

#### 2.2.1. Developmental features.

**Fig 2:**
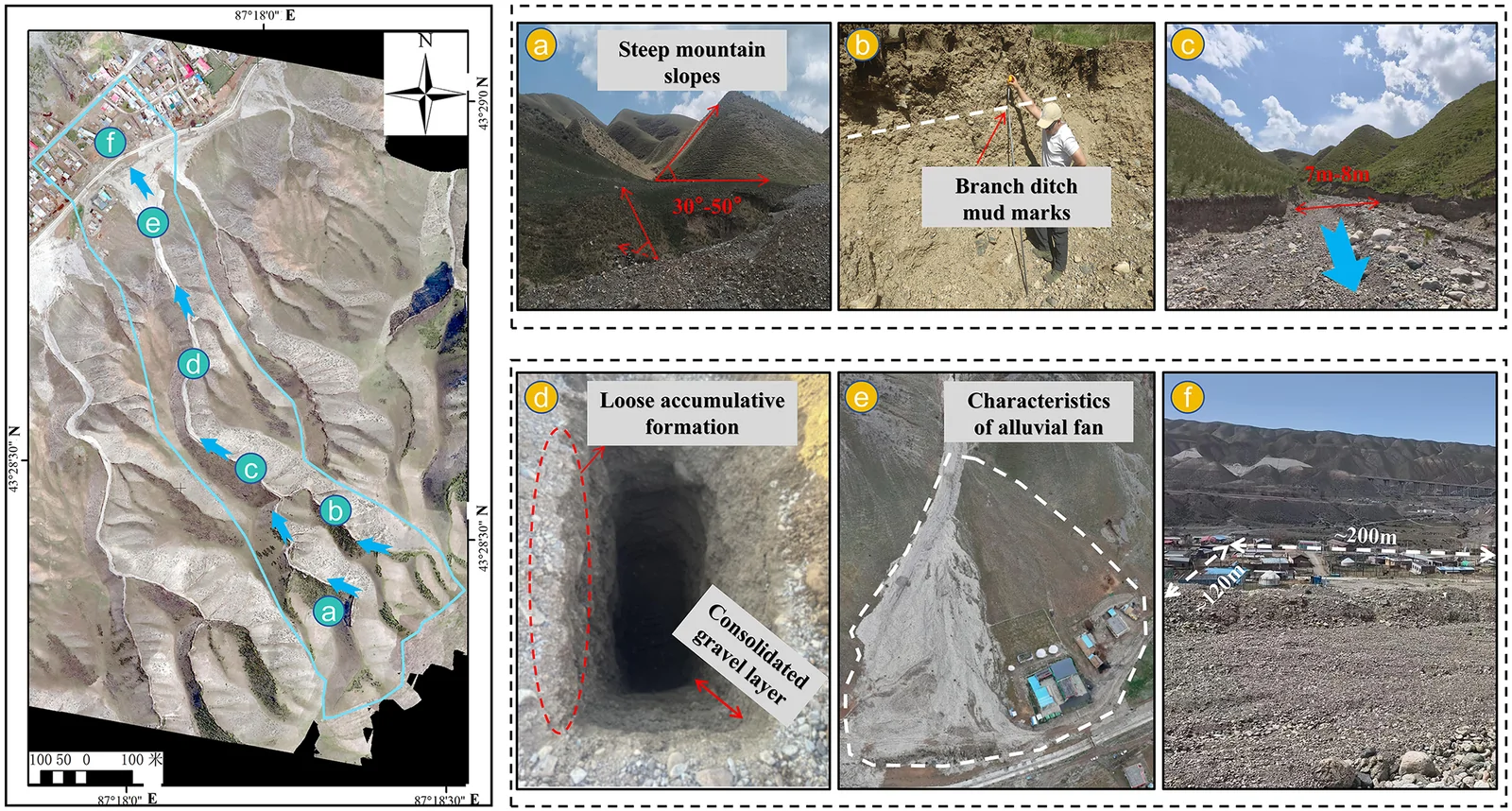
Geomorphological and developmental characteristics of the debris flow watershed. (The base map was generated by the authors using field UAV survey data.All photographs and experimental graphs in this figure were captured and generated by the authors during field investigations and laboratory testing.).

This study focuses on a representative steep-gully debris flow located approximately 65 km from Urumqi, which exhibits the maximum longitudinal gradient in the region. The drainage basin covers 0.35 km², categorized into a formation area (0.15 km²), a transportation area (0.13 km²), and a deposition area (0.07 km²). The main gully extends 1.45 km with a total relief of 289.8 m. The average longitudinal gradient is 266.3‰, peaking at 330.5‰ in the formation area. The gully displays a “Y-shaped” planar morphology and a broom-shaped watershed, with cross-sections transitioning from “V-shaped” to “U-shaped” from south to north (Fig 2).

The debris flow outbreaks are driven by the synergy of steep topography (potential energy), loose lithology (material source), and extreme hydrology (triggering source). Field investigations reveal that the gully possesses a high watershed slope and deep incision. The wide transportation zone within the formation area promotes rainwater capture, while landslide and collapse deposits on the steep flanking slopes (30°–50°) provide ample solid material (Fig 2a). Mud marks (Fig 2b) and traces in branch gullies indicate high transport capacity. Despite relatively high vegetation coverage, the dominance of herbaceous plants offers negligible mechanical reinforcement to the gully slopes.

The transportation zone is characterized by a narrow, straight channel (7–8 m wide) with a high longitudinal gradient (Fig 2c). A distinctive binary structure—loose surface deposits overlying consolidated sandy gravel—facilitates rapid acceleration of the flow under gravity (Fig 2d). The deposition zone at the gully mouth forms a trumpet-shaped alluvial fan (Fig 2e), consisting of loose silt, sand, and pebbles (0.05–0.2 m in size). Recent activity shows that debris flows can surmount existing dry canals at the gully mouth, directly threatening residential areas (Fig 2f).

Debris flows in this region exhibit pronounced seasonality, primarily concentrated in June and July. During the dry season, the gullies remain largely inactive. However, short-term intense rainfall during the monsoon triggers rapid hydro-dynamic concentration, enabling the flow to mobilize massive volumes of loose slope and gully deposits instantaneously.

#### 2.2.2. Engineering geological characteristics of the watershed.

The upper and middle reaches of the debris flow catchment feature remarkably steep topography, with bank slope gradients typically ranging from 30° to 50°. In these high-elevation zones, the surface cover is sparse and relatively thin. Conversely, the middle and lower reaches exhibit increased cover thickness and a broader distribution of loose materials on the steep bank slopes. During intense rainfall, the rapid convergence of surface runoff generates high-velocity torrential flows with significant dynamic force. These flows entrain slope-wash and loose gully deposits, surging downstream to initiate debris flows. The relatively straight gully alignment and minimal siltation in the formation area prevent energy dissipation, allowing the hydraulic power to concentrate. This concentration exacerbates bank collapse and provides the necessary geo-environmental conditions for debris flow mobilization. The distribution of rock and soil masses within the watershed is detailed below:

1. Distribution of rock masses

**Fig 3:**
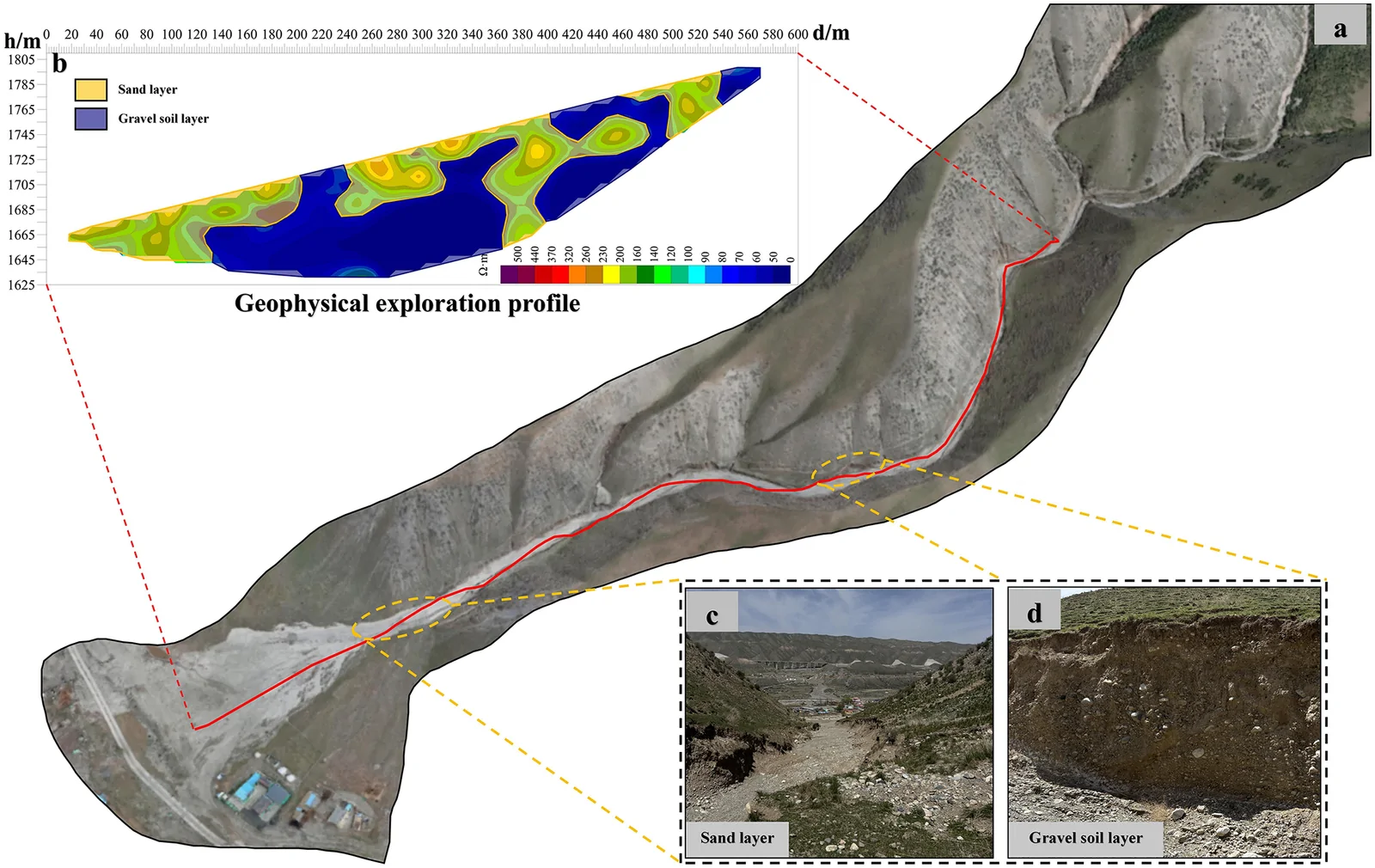
Spatial distribution of engineering geological formations in the study area. The base map was generated by the authors using field UAV survey data.All photographs and experimental graphs in this figure were captured and generated by the authors during field investigations and laboratory testing.

The exposed strata primarily consist of Permian sedimentary rocks, predominantly distributed in the formation area (Fig 3a). The dominant lithologies are conglomerate and glutenite. The surface layers of these rock masses are intensely weathered, with well-developed joints and fissures resulting in a blocky, fragmented structure. Typical block sizes range from 0.03 to 0.2 m, with larger fragments reaching 0.3 to 0.6 m. These rocks are calcareously cemented and relatively hard, exhibiting a medium-to-fine grained texture and a layered structure. Due to intense surface weathering, the rock masses present moderate engineering geological properties.

2. Distribution of soil masses

The Quaternary Holocene deposits in the study area include slope-wash, alluvial, and proluvial sediments, categorized into two distinct layers based on their genesis and lithology (Fig 3b). Geophysical surveys indicate that the soil composition is primarily gravelly, comprising silty sand, crushed stone, and pebbles. These are classified as follows:

① Sand Layer (Fig 3c): The upper sequence consists of a taupe, loess-like sand layer. It exhibits a loose-to-dense texture and ranges from dry to slightly moist. A vegetative root zone is developed within the shallow surface (0.2–0.5 m), and the total layer thickness varies between 1 and 2 m.

② Gravel and Cobble Layer (Fig 3d): The lower sequence is dominated by grayish-white, semi-cemented gravel and cobbles. The clasts (crushed stone and pebbles) exhibit poor-to-moderate roundness, with diameters generally between 5 and 15 cm. Coarse clastics account for 60%–70% of the total mass. This layer is characterized by its significant and uniform thickness, exceeding 10 m. The lithology of the clasts includes coarse sandstone, glutenite, siltstone, and gravelly clastic limestone, forming a loose-to-slightly dense structure.

The stratigraphic sequence within this steep-gully watershed is relatively straightforward, dominated by thick alluvial-proluvial deposits. Although the thick, layered gravel and cobble masses provide generally stable engineering geological conditions for infrastructure, the deeply incised gully sections remain highly susceptible to providing abundant material sources for debris flow activity.

#### 2.2.3. Material source supply characteristics.

The solid material sources of the steep-gully debris flow in the study area are primarily categorized into loose surface materials on the flanking slopes and deposits within the gully bed. Field investigations reveal that the Quaternary cover on the slopes is extensive, with a thickness ranging from 1 to 3 m, providing a sustained supply of solid material. Within the gully channel, solid sources are predominantly derived from loose bed deposits (Fig 3c), with gully bank erosion serving as a secondary contributor (Fig 3d). The dynamic mobilization is primarily driven by hydraulic scouring and undercutting of the gully bed; these materials are highly susceptible to being entrained and transformed into debris flow sources during intense rainstorms.

The participation of gully bed deposits in debris flow activity is primarily through basal scouring. While the volume mobilized under normal hydrological conditions is relatively small, extreme rainstorms drastically enhance the hydrodynamic conditions. This leads to intensive scouring and vertical undercutting, entraining vast quantities of bed material to form large-scale, highly destructive debris flows.

#### 2.2.4. Characteristics of debris flow deposits.

The alluvial fan at the terminus of the steep-gully debris flow remains relatively intact, extending approximately 330 m in length and 270 m in width, with a divergence angle of about 90° and a total area of 0.074 km². The deposits consist of gravel accumulations matrix-filled with sandy soil. The deposition area maintains a relatively steep gradient of approximately 15°–20°. Geophysical prospecting indicates a deposit thickness ranging from 5 to 40 m, exhibiting a general coarsening-upward or longitudinal grading from fine to coarse. Large cobbles are primarily concentrated at the fan’s leading edge, while sandy soil and fine-grained materials fill the interstices, creating a loose structure with distinct sorting features.

**Fig 4:**
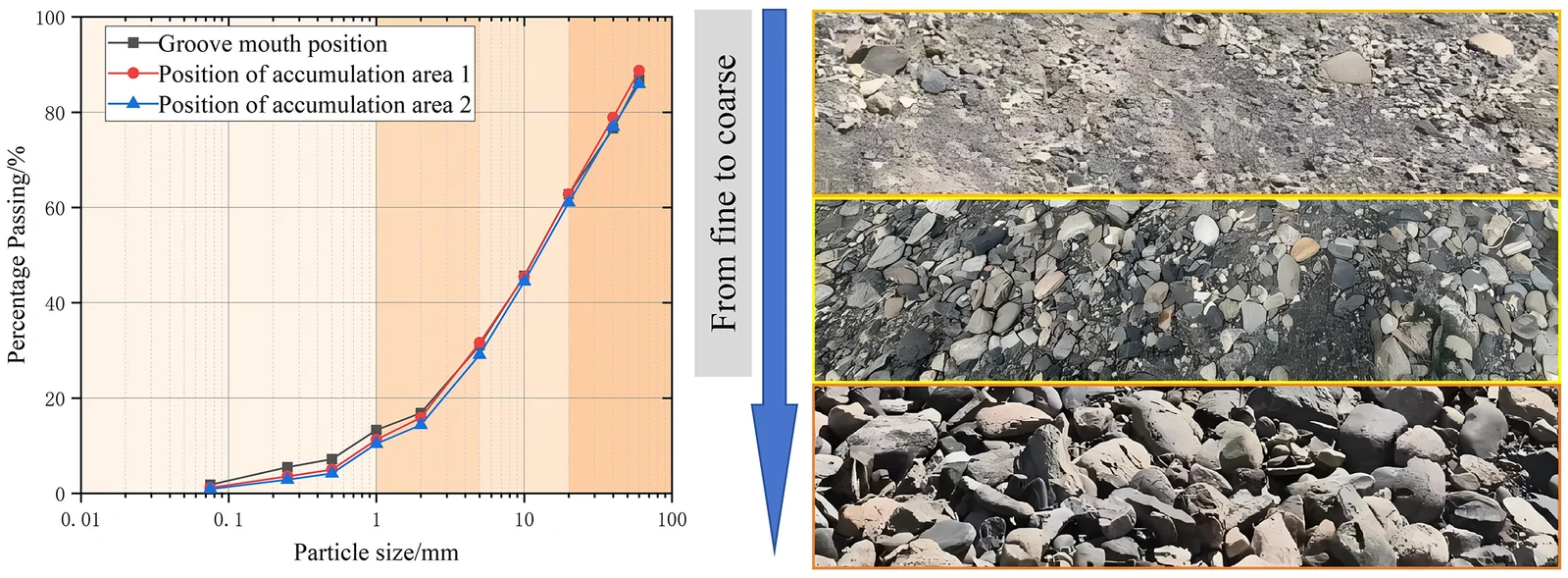
Grain size distribution and gradation characteristics of the gully mouth and deposition zones. All photographs and experimental graphs in this figure were captured and generated by the authors during field investigations and laboratory testing.

The deposits are dominated by cobbles and gravels of varying sizes. Particle size distribution (PSD) analysis of field samples indicates poor sorting, with grain sizes ranging from several millimeters to tens of centimeters; however, the overall gradation is continuous and well-graded (Fig 4). The surface morphology of the deposition area is complex, reflecting the cumulative effects of multiple debris flow events and prominent late-stage scouring traces.

At the gully mouth, the gradation curve is relatively gentle, reflecting a continuous particle size distribution and high-quality gradation. In contrast, the slopes of the curves for Deposition Zones 1 and 2 increase significantly, indicating an enrichment of coarse particles and a more concentrated grain size distribution (Fig 4a). The variation from fine to coarse particles from the gully mouth to the outer fan (Fig 4b) suggests that flow energy gradually attenuates during runout. Consequently, larger particles settle preferentially, while finer materials continue to be transported, resulting in a spatial pattern of uniform gradation at the gully mouth and coarse-particle enrichment in the distal deposition zones.

## 3. Analysis of formation conditions and disaster mechanisms

The study area is situated in the arid region of Northwest China, specifically within the central section of the northern Tianshan Mountains. Recently, global warming and the increasing frequency of extreme weather events have significantly impacted the Xinjiang region—the heart of Asia [36]. This has resulted in a northward shift of the regional rain belt, leading to more concentrated precipitation along the northern slopes of the Tianshan Mountains [37]. Consequently, both the frequency and intensity of extreme rainfall events have increased, providing more frequent triggering conditions for debris flow initiation. In steep-gully environments characterized by rugged terrain and abundant source material, these shifts significantly heighten the hazard of debris flow mobilization.

A major debris flow event occurred in the study area in July 2024. This sudden disaster caused extensive structural damage to residential buildings, including structural tilting, widespread wall cracking, and severe deformation of doors and windows. Massive accumulations of rock and sediment partially buried buildings, obstructing access. Infrastructure was similarly affected: several road sections were washed away or buried, leading to traffic disruption, and utility facilities such as electric poles were destroyed. Total economic losses were estimated to exceed 5 million yuan.

### 3.1. Rainfall conditions

Precipitation in the study area is primarily concentrated in June and July, occurring predominantly as short-duration, high-intensity rainstorms. The substantial volume of rainfall facilitates rapid surface runoff generation. Groundwater in the area consists mainly of pore water within Quaternary loose rocks. The aquifer, composed of a single layer of sandy gravel, exhibits high porosity and high permeability. Furthermore, the steep and narrow geometry of the gully channel causes flow to accelerate continuously under gravity, leading to rapid hydro-dynamic concentration and the formation of high-energy torrential flows.

**Fig 5:**
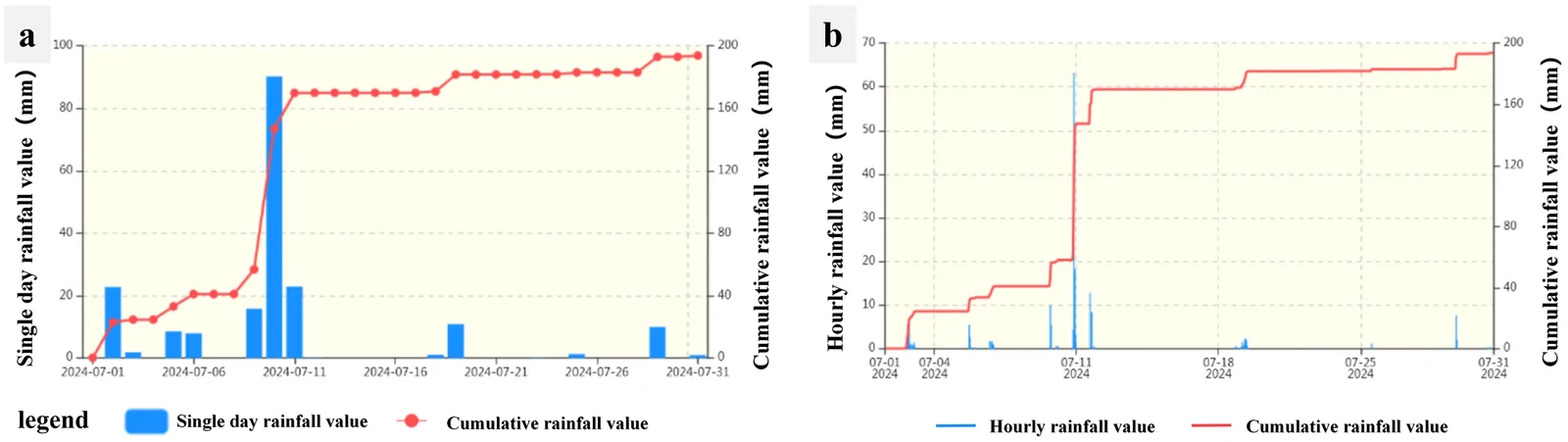
Temporal distribution of daily and peak hourly rainfall during the July 2024 debris flow event.

The debris flow channel is heavily influenced by seasonal orographic rainfall, resulting in significant annual discharge fluctuations. According to local rain gauge monitoring, the daily rainfall on the day of the 2024 event was 90.2 mm (Fig 5a), with the peak hourly intensity reaching 63.2 mm (Fig 5b). These data underscore the typical characteristics of short-duration, high-intensity rainfall as the primary triggering factor.

A comparative analysis of the daily and peak hourly rainfall variations reveals a distinct single-peak concentration pattern throughout the precipitation event. The cumulative rainfall exhibited a stepwise increase, characterized by a sharp surge on the day of the primary rainstorm. The peak hourly intensity on July 11 underscores the episodic, short-term burst nature of the event, after which rainfall gradually subsided until the cumulative volume stabilized. This temporal evolution confirms that short-duration, high-intensity rainfall was the decisive trigger for the debris flow.From a temporal perspective, the highly non-uniform spatiotemporal distribution of precipitation in the study area significantly exacerbated the hazard risk. The rapid accumulation of high-volume precipitation within a confined timeframe facilitated the instantaneous generation and concentration of surface runoff, providing the necessary hydrodynamic power and water volume to initiate the debris flow.

### 3.2. Material source conditions

#### 3.2.1. Characteristics of the displacement.

**Fig 6:**
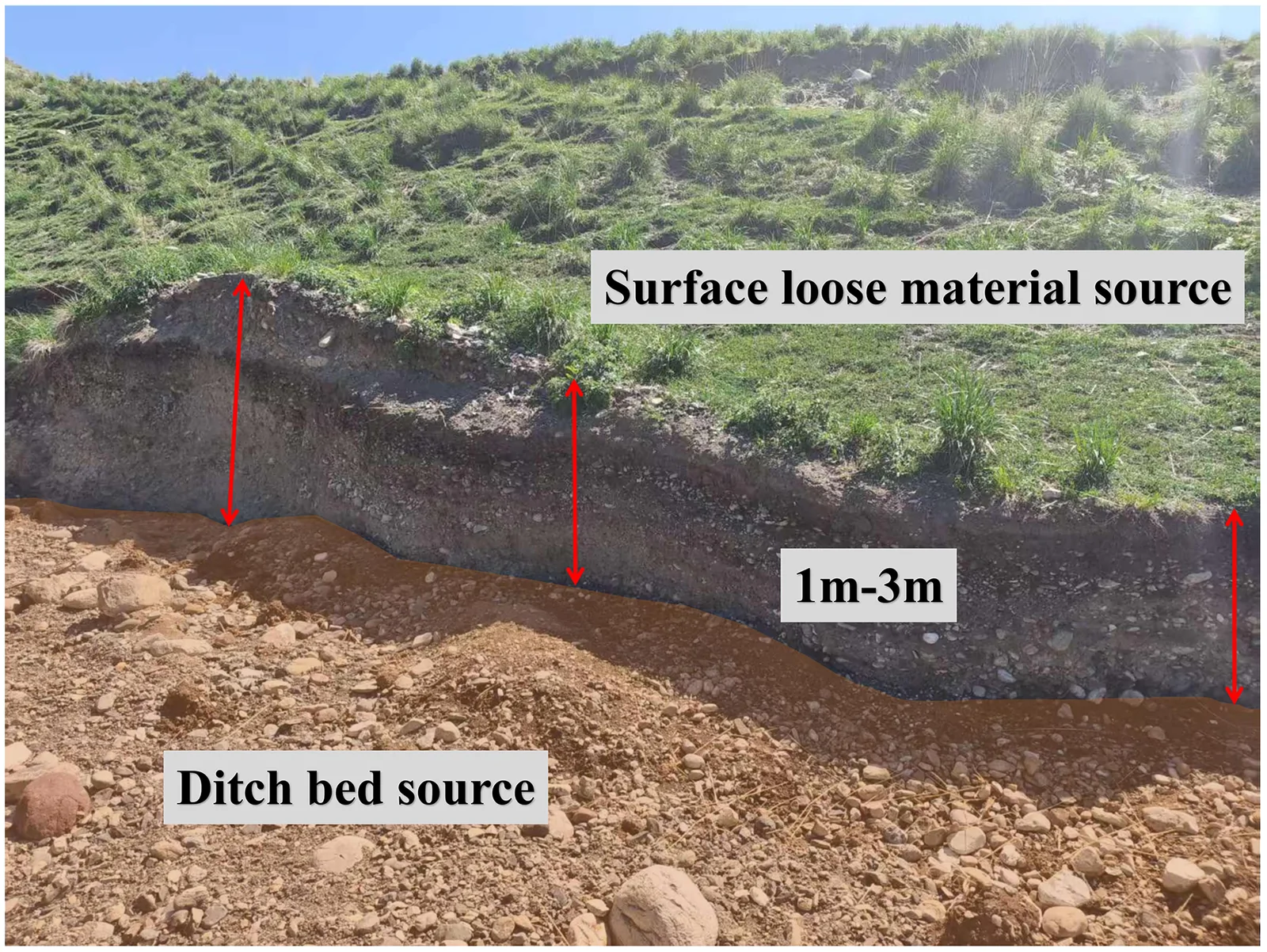
Characteristics of material sources for the debris flow disaster in the study area. All photographs and experimental graphs in this figure were captured and generated by the authors during field investigations and laboratory testing.

Debris flow material sources in the study area are categorized into two types: gully bed deposits and loose slope-surface materials. The latter are distributed within the shallow surface layer (1–3 m) of the flanking slopes, concentrated on gradients ranging from 30° to 50° (Fig 6). This material consists primarily of residual eluvial soil interspersed with plant roots, characterized by a loose structure and high porosity. Subjected to both rainfall infiltration and the mechanical reinforcement of root systems, these deposits serve as the primary medium for slope sheet erosion. Under intense rainfall, these source materials are highly susceptible to shallow sliding or localized collapse, providing a continuous sediment supply to the gully.Gully bed material sources are predominantly distributed along the main and branch channels (Fig 6). These consist of Quaternary alluvial-proluvial sandy gravel layers, with borehole-verified thicknesses of 5–8 m. The material composition is dominated by poorly rounded cobbles and gravel, with interstices filled by fine-to-coarse sand. Due to long-term hydraulic scouring, this layer exhibits a loose structure and high permeability. Under extreme rainfall, the bed is prone to intensive basal scouring, resulting in downcutting depths of 2–3 m, which subsequently triggers lateral bank collapse.Field investigations of the 2024 debris flow event reveal that mobilized gully bed materials accounted for approximately 60% of the total volume. Notably, lateral collapses induced by bed downcutting contributed 40% of this volume. This underscores that the activation of gully bed materials is the dominant factor driving the scale expansion of debris flows in this catchment. Statistical analysis indicates that the total reserve of loose solid material in the study area is approximately 2.19 × 10^4^m^3^, with a maximum dynamic reserve (movable in a single event) of approximately 0.67 × 10^4^m^3^.

### 3.3. Analysis of the disaster-triggering mode

**Fig 7:**
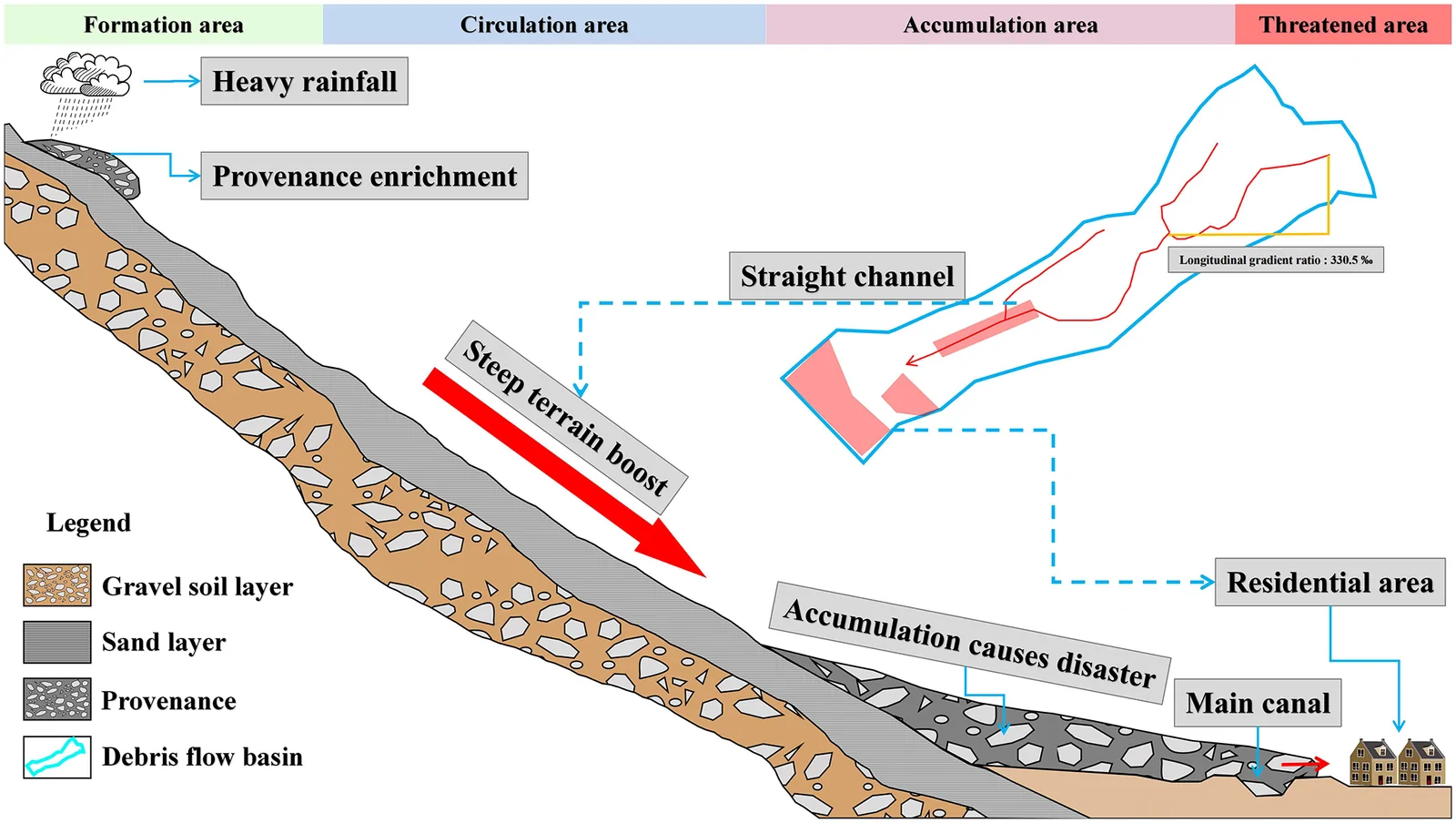
Disaster-causing mode diagram of steep gully debris flow in the study area. All clip art and schematic vectors are sourced from open-source public domains (https://openclipart.org/).

Debris flows are widespread geological hazards globally [38], with their disaster-triggering modes exhibiting diverse characteristics governed by regional geological settings, climatic conditions, and material source properties. In the study area, the steep-gully debris flow features a unique disaster mode resulting from the long-term coupling of topography, lithology, meteorology, and tectonic structures. This study defines the process as a “Source Enrichment – Dynamic Triggering – Path Transmission – Accumulation-Induced Hazard” chain. The core driving mechanism is the synergy of “loose material enrichment + intense rainfall triggering + steep terrain propelling.” Ultimately, the debris flow surges through the channel and impacts the deposition area, threatening the irrigation canal and residential settlements. These interconnected stages constitute a sequential and integrated disaster-triggering system (Fig 7).

1. Topographic and geological foundations

The study area is located in the northern Urumqi River system, characterized by deeply incised mid-low mountain geomorphology. The terrain slopes from south to north with altitudes ranging from 1,560 m to 1,930 m. The average longitudinal gradient of the main gully is 266.3‰, with gradients in the formation and transportation zones reaching 330.5‰ and 248.5‰, respectively. Bank slopes range from 30° to 55°, providing a formidable gravitational potential energy base for debris flow activity.

The lithology is dominated by intensely weathered Permian sedimentary rocks (conglomerates and glutenites) alongside Quaternary Holocene alluvial-proluvial and eluvial deposits. Soft formations, such as silty and breccia soils, are widespread on the surface. Furthermore, proximity to a seismically active zone has exacerbated rock fragmentation and debris accumulation. The Quaternary cover on the gully bed and slopes is 1–3 m thick. With two active branch gullies and extensive landslide and collapse deposits flanking the main channel, the watershed maintains an abundant and easily mobilized material reserve, fulfilling the criteria for source enrichment.

2. Meteorological triggering and hydro-dynamic mechanisms

Meteorological conditions serve as the decisive trigger, where intense rainfall coupled with topographic amplification creates a highly efficient dynamic driving system. Summer rainfall (June–August) accounts for 32.6%–73.3% of the annual total, predominantly occurring as rainstorms. During the 2024 event, a daily rainfall of 90.2 mm and a peak hourly intensity of 63.2 mm were recorded—typical of short-duration, high-intensity rainfall that can rapidly converge into high-magnitude torrential flows.

The Y-shaped gully geometry and broad formation zone facilitate efficient runoff capture. In the transportation zone, the narrow (7–8 m) and straight channel concentrates hydraulic energy and prevents dissipation. The gully’s binary structure—loose surface deposits overlying consolidated sandy gravel—restricts infiltration and promotes instantaneous runoff concentration and acceleration. Under the impulse of intense rainfall and steep terrain, flow velocities surge, significantly enhancing bed scouring and slope erosion. This rapidly mobilizes loose deposits into a seasonal debris flow, active during the June–July rainy season and dormant during the dry season.

3. Path transmission and evolutionary features

Once formed, the debris flow advances rapidly along the channel. Given the high gradient of the formation zone (330.5‰), the flow possesses immense potential energy at its inception. It maintains this energy and transports solid material through path transmission. The straight, narrow channel and high longitudinal gradient in the transportation zone provide the debris flow with exceptional velocity and carrying capacity. Despite high vegetation coverage, the dominance of herbaceous plants offers limited mechanical reinforcement and fails to impede the high-energy flow.

4. Accumulation and disaster impact

The deposition area features a trumpet-shaped alluvial fan with a gradient of 15°–20°. Deposits are primarily composed of cobbles and gravels mixed with sand, showing pronounced spatial sorting: large cobbles concentrate at the fan front, while fines fill the interstices, resulting in a loose structure with thicknesses of 5–40 m.

The disaster follows the sequence of “Channel Transport – Canal Sedimentation – Residential Threat.” Upon exiting the gully, the debris flow first infills the primary irrigation canal with sediment. Subsequent surges then overtop the canal and directly impact downstream residential areas. Furthermore, as energy attenuates from the gully mouth to the outer fan, a spatial pattern of “uniform gradation at the mouth and coarse-particle enrichment in the deposition area” emerges. Repeated activity has complicated the surface morphology and elevated the local terrain, creating new topographic conditions and material sources for future disasters.

The disaster-triggering mode of the steep-gully debris flow in the study area is the result of multi-field coupling involving topography, lithology, hydro-meteorology, and geological structures. This synergy constitutes a comprehensive disaster chain characterized by “material source enrichment – dynamic triggering – path transmission – accumulation-induced hazard.”

**Fig 8:**
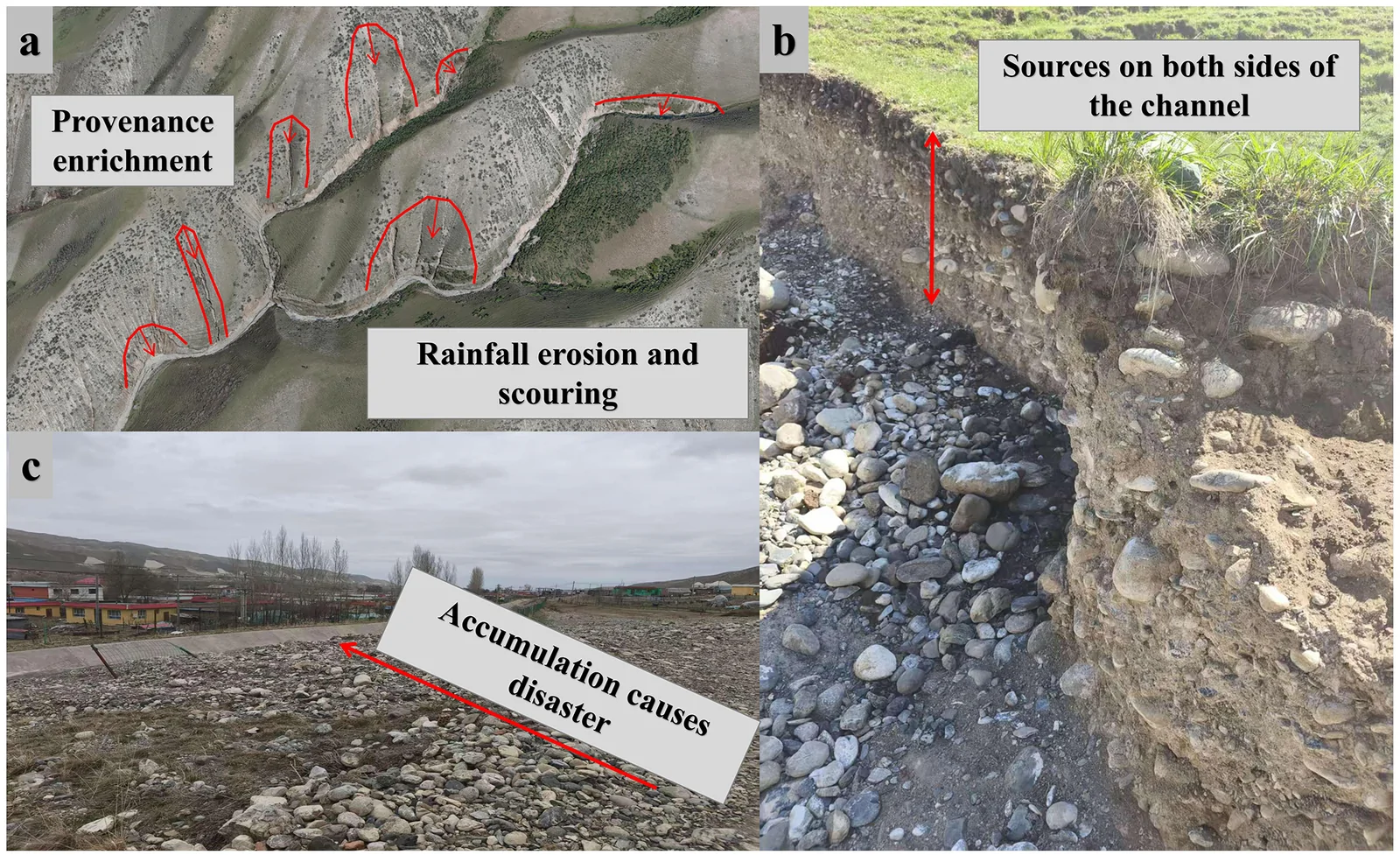
Evolutionary process and sequential stages of the disaster-triggering mode. The base map was generated by the authors using field UAV survey data.All photographs and experimental graphs in this figure were captured and generated by the authors during field investigations and laboratory testing.

The catchment features deeply incised mid-low mountain geomorphology with a pronounced south-to-high terrain gradient. The average longitudinal gradient of the main gully reaches 266.3‰, while bank slopes range from 30° to 55°. Highly weathered Permian sedimentary rocks and Quaternary loose deposits are extensively distributed. The superimposed effect of seismic activity has further intensified rock mass fragmentation, creating an abundant and highly mobile material reservoir for debris flow initiation (Fig 8a).

Short-duration, high-intensity summer rainfall—accounting for 32.6%–73.3% of the annual total—serves as the primary triggering dynamic force. Rainwater undergoes rapid hydro-dynamic concentration through the Y-shaped gully network and the binary-structured gully beds. Under the topographic amplification of the steep channels, loose materials are instantaneously mobilized (Fig 8b), forming high-energy seasonal debris flows.

The debris flow is transported efficiently along the straight and narrow transportation zone, maintaining high kinetic energy until it impacts and diffuses across the trumpet-shaped alluvial fan. The resulting deposits are characterized by a loose structure dominated by cobbles and gravel. Upon exiting the gully mouth, the debris flow typically infills the primary irrigation canal before overtopping it to threaten downstream residential areas (Fig 8c), completing the disaster evolution process.

## 4. Numerical simulation of steep-gully debris flow using FLO-2D

### 4.1. Model principles and simulation parameters

In this study, the FLO-2D numerical simulation software was employed to perform a dynamic analysis of debris flow behavior. FLO-2D is a sophisticated two-dimensional hydrodynamic model extensively applied to simulate the evolution and propagation of natural hazards, including floods and debris flows [39–41]. The governing equations, originally proposed by O’Brien [42], have been progressively optimized based on non-Newtonian fluid rheology and the central finite difference method. It is currently a standard tool for solving solid-liquid two-phase flow problems [43]. The model discretizes the computational domain into regular or irregular grid cells, calculating key parameters such as flow depth and velocity within each cell to simulate debris flow kinematics under complex topographic conditions.

### 4.2. Rainfall selection and boundary conditions

The steep-gully debris flows in the study area are primarily triggered by short-duration, high-intensity rainfall. To capture this accurately, on-site rain gauges were utilized. As previously established, a daily rainfall of 90.2 mm was recorded during the 2024 event, with a peak hourly intensity of 63.2 mm. These high-fidelity data, combined with historical rainfall threshold analysis, were selected as the typical boundary conditions for this simulation.

**Fig 9:**
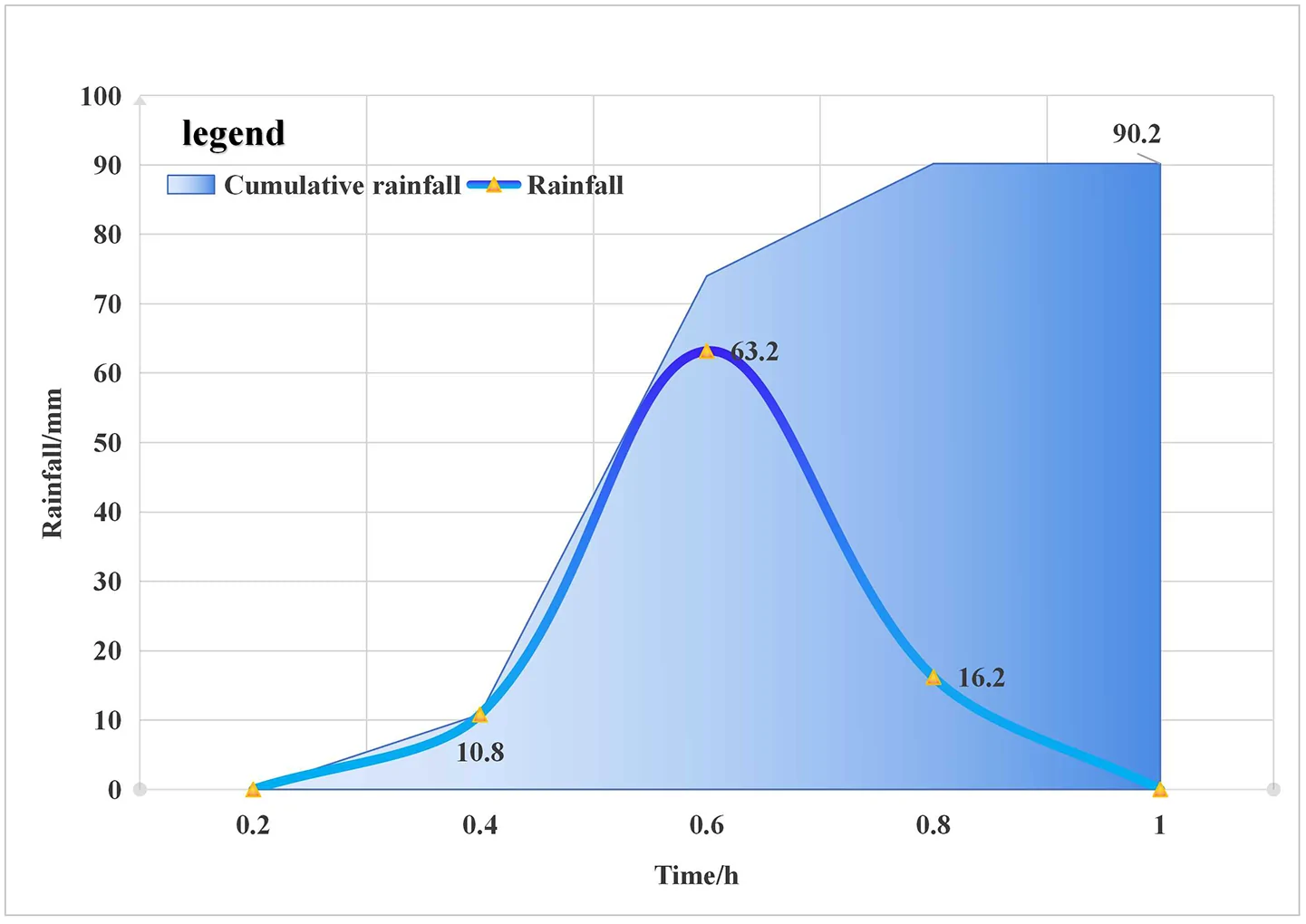
Daily cumulative rainfall process diagram of the study area.

The rainfall station is located in the middle-to-upper reaches of the main gully within the target watershed, serving as the core controlling station for debris flow hazard monitoring. The recorded data accurately characterizes the actual rainfall patterns within the catchment. Field investigations and on-site rain gauge monitoring data indicated that the triggering rainfall occurred exclusively during a one-hour window in the afternoon. To ensure the simulation aligns with actual observations, the 90.2 mm daily total and 63.2 mm hourly peak were synthesized into a continuous one-hour time series. This rainfall process was then discretized at 0.2-hour intervals to provide high-resolution input for the numerical model (Fig 9).

### 4.3. Parameter determination and calibration

The selection of parameters for the FLO-2D simulation was informed by field investigations, empirical values from the FLO-2D user manual, and a comprehensive review of literature concerning debris flow characteristics along the northern slopes of the Tianshan Mountains. The final dataset includes critical variables such as bulk density, sediment volume concentration, Manning’s roughness coefficient, laminar flow resistance, yield stress, dynamic viscosity, and the precipitation amplification factor.

1. Debris flow bulk density and volume concentration

Based on particle size distribution (PSD) analysis, in-situ bulk density tests, slurry proportioning experiments, and the physical characteristics of residual mud marks, it was determined that the debris flow exhibited a “dilute slurry” state during the peak of the event. The natural bulk density of the loose deposits in the deposition area (comprising rounded gravel and sand) is 2.52 g/cm³. For the solid phase within the fluid, the average test value of 1.76 g/cm³ was adopted. Consequently, the sediment volume concentration (Cv) was calculated to be 50%.

2. Laminar flow resistance coefficient

The laminar flow resistance coefficient (K) quantifies the surface resistance exerted against the debris flow during its movement. This value is sensitive to the type, scale, and density of surface vegetation. While the standard range for K spans from 24 to 50,000, a comprehensive value of K = 2220 was determined for this study using the engineering geological analogy method and standard lookup tables.

3. Rheological parameters

The yield stress of a debris flow is a critical threshold; the fluid transitions from a stable state to a flowing state (instability) only when the internal shear stress exceeds this value. Both the yield stress (τy) and the dynamic viscosity coefficient (η) exhibit a strong exponential correlation with the sediment volume concentration. The relationships between volume concentration and these rheological parameters are expressed as follows:


$$\begin{array}{c} \eta =\alpha_{\text{1}}e^{\beta_{\text{1}}C_{v}} \end{array}$$ (1)

$$\begin{array}{c} \tau_{y}=\alpha_{\text{2}}e^{\beta_{\text{2}}C_{v}} \end{array}$$ (2)

where [α1, β1, α2, β2] are empirical coefficients, which are obtained from the FLO-2D manual as [0.811, 0.0046, 13.72, 11.24] respectively. Through calculation, the values of [η,τy] are determined as [0.0081, 17.92].

4. Manning roughness coefficient

The Manning’s roughness coefficient (n) represents the frictional resistance exerted by the gully bed and banks on the debris flow. In this study, the value of n was determined following the empirical formula for debris flow resistance proposed by Wang Yuyi et al [44].


$$\begin{array}{c} n=\;\text{0}.\text{033}R_{ns}^{-\text{0}.\text{51}}{exp}^{\text{0}.\text{34}R_{ns}^{\text{0}.\text{17}}}\text{ln}\text{h} \end{array}$$ (3)

where:h——mud depth/m; exp——average void ratio.

Given that the Manning’s coefficient (n) is a dynamic variable, an average flow depth of 1.61m within the debris flow watershed was adopted for the calculation. Based on the recommended parameter ranges provided in the FLO-2D technical manual—accounting for the approximately 20% vegetation coverage in the study area (with a reference range of 0.05–0.25)—and integrated with field survey data, the Manning’s coefficient was comprehensively determined to be 0.0774.

5. Peak discharge calculation

In this study, the FLO-2D model was utilized to simulate debris flow dynamics under three distinct scenarios: the historical event of July 2024, a 50-year return period (P = 2%), and a 100-year return period (P = 1%). For the 2024 event, the peak discharge was calculated using the rational formula method driven by on-site measured rainfall data, in accordance with the rainstorm research for small watersheds in Xinjiang as documented in the Atlas of Design Rainstorm and Flood in Small Watersheds of Xinjiang Uygur Autonomous Region. The specific empirical formula tailored for mountainous small watersheds is as follows:


$$\begin{array}{c} Q_{p}\;=\text{0}.\text{2}\text{7}\text{8}\Psi \text{iF} \end{array}$$ (4)

In the rational formula, Ψ represents the peak runoff coefficient, which is assigned a value of 0.7 based on the surface cover characteristics of the study area; i is the maximum hourly rainfall intensity, recorded at 63.2 mm/h; and F is the watershed catchment area, measuring 0.35 km². Under these parameters, the peak discharge for the 2024 scenario is calculated to be 4.30 m³/s.

For the 50-year (P = 2%) and 100-year (P = 1%) return period scenarios, the Xibaiyanggou Station, located 5 km west of the study area, was selected as the hydrological reference station. The peak discharges for these design frequencies were calculated using the modulus coefficient method, according to the following formula:


$$\begin{array}{c} Q_{PS}=({Fs/\text{Fc})}^{\text{n}}Q_{PC} \end{array}$$ (5)

where:Q_PC_—Design peak discharge of the reference station(m^3^/s),50-year return period discharge=33.66, 100-year return period discharge=50.44;

F_C_—Catchment area of the reference station(km^2^)=15.4;

Q_PS_—Design peak discharge at the calculation cross-section(m^3^/s);

F_S_—Design catchment area of the calculation cross-section(km2)=0.35;

n—Area ratio exponent factor=0.5.

By substituting the respective parameters into the formula, the peak discharges for the study area under the 50-year (P = 2%) and 100-year (P = 1%) return period scenarios were determined as Qps 50 = 5.07 m^3^/s,Qps 100 = 7.60 m^3^/s, respectively.

To ensure the reliability of these calculated values, a cross-verification was performed using the area-ratio comparison method, integrated with field survey data from adjacent watersheds with similar geomorphological characteristics. The results showed that the deviations were consistently below 8%, confirming that the calculated peak discharges are highly representative and suitable for the subsequent numerical simulation.

### 4.4. Analysis of numerical simulation results

The baseline topographic data in this study employed a multi-source fusion strategy. The macro-watershed boundaries and hydrological features were extracted using a 12.5 m resolution ALOS PALSAR DEM obtained from ASF DAAC. For the main debris flow gully and the hazardous runout zones, an ultra-high-resolution DEM with a spatial resolution of 0.15 m was constructed using field surveys and Unmanned Aerial Vehicle (UAV) oblique photogrammetry. During the numerical simulation phase, to balance computational efficiency and topographic detail, the fused baseline terrain was resampled and discretized into a uniform computational grid of 5 m × 5 m. Based on field survey data and the measured deposition footprint of the 2024 debris flow event, the rheological parameters of the model were first back-calculated and validated within this 5 m grid framework. Subsequently, the calibrated FLO-2D model was utilized to conduct forward-looking numerical simulations of debris flow dynamic characteristics and hazard zonation under the 50-year and 100-year extreme rainfall scenarios.

To ensure data interoperability, the DEM was processed within the ArcGIS platform and converted into ASCII format to meet the requirements of the FLO-2D computational engine. The computational domain was discretized into a 5m × 5m grid. Subsequently, the calibrated rheological and Manning parameters were assigned, and the inflow hydrograph was applied as the boundary condition. The simulation analyzed the velocity, flow depth, and hazard intensity under three scenarios: the 2024 historical event, the 50-year return period (P = 2%), and the 100-year return period (P = 1%).

#### 4.4.1. Model validation and analysis: The 2024 scenario.

**Fig 10:**
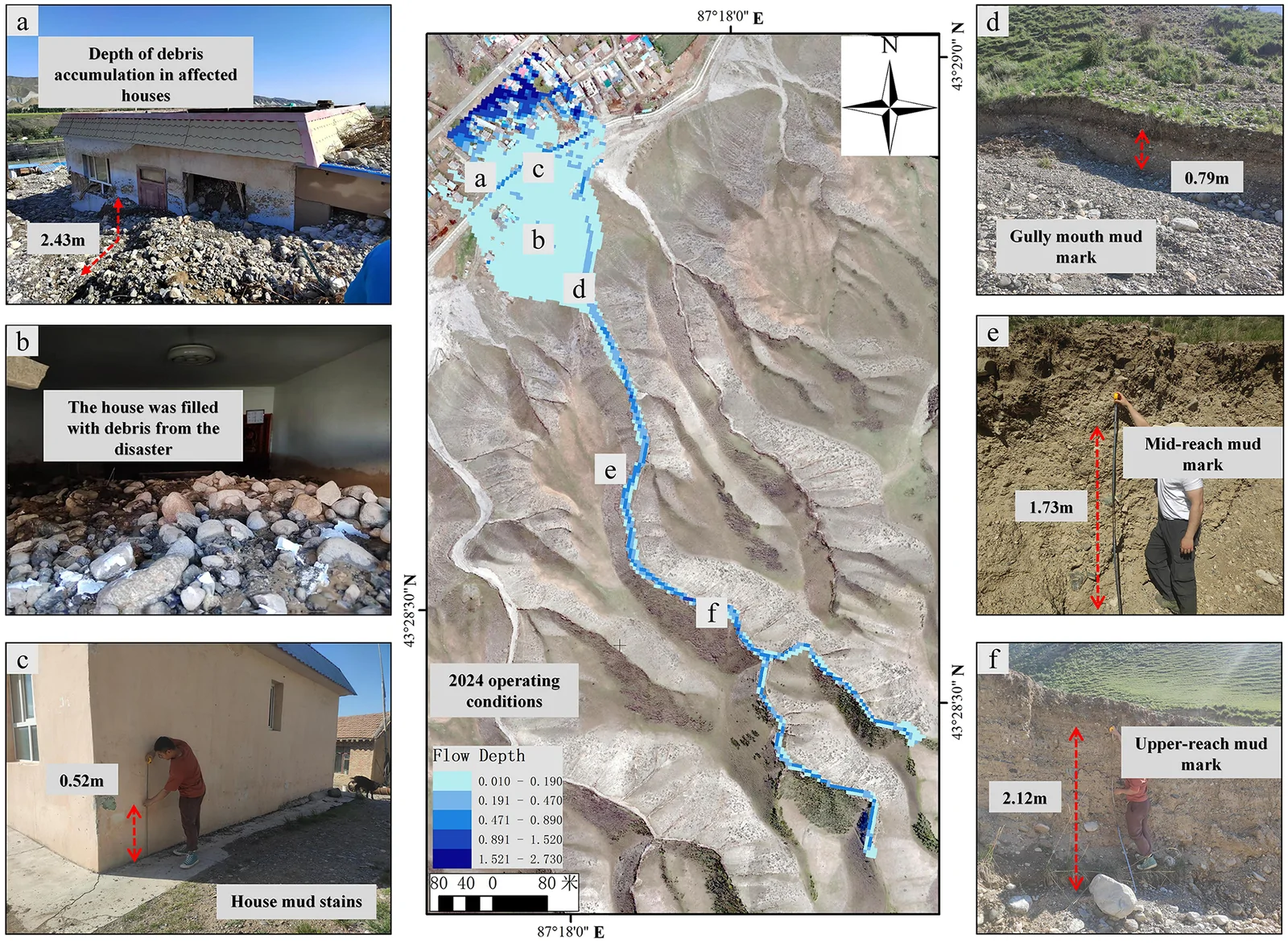
Validation of simulated debris flow depth against field measurements for the 2024 scenario. (The base map was generated by the authors using field UAV survey data.All photographs and experimental graphs in this figure were captured and generated by the authors during field investigations and laboratory testing.).

Validation of the 2024 scenario was conducted by cross-referencing simulation outputs with on-site disaster investigations and mud mark (high-water mark) measurements. The simulated maximum flow depth reached 2.73m, localized at the residential structures within the accumulation area—a result closely aligning with the post-disaster measured depth of 2.43m (Fig. 10a).

In terms of spatial distribution, the flow depth in the upper reaches of the accumulation zone was relatively shallow (Fig 10b), averaging approximately 0.5m (Fig 10c). This phenomenon is attributed to the steep geomorphological characteristics of the gully; the high longitudinal gradient facilitated high-velocity transport and minimized localized deposition within the channel. Significant accumulation only occurred upon reaching the residential areas, where the flow transitioned and silted.From the formation to the transportation zones, the simulated mud marks exhibited a progressive decrease in depth (Fig 10f → d), consistent with the dynamic attenuation of the debris flow along its trajectory. The narrow, straight channel geometry constrained the fluid, leading to the partial deposition of solid materials.

**Fig 11:**
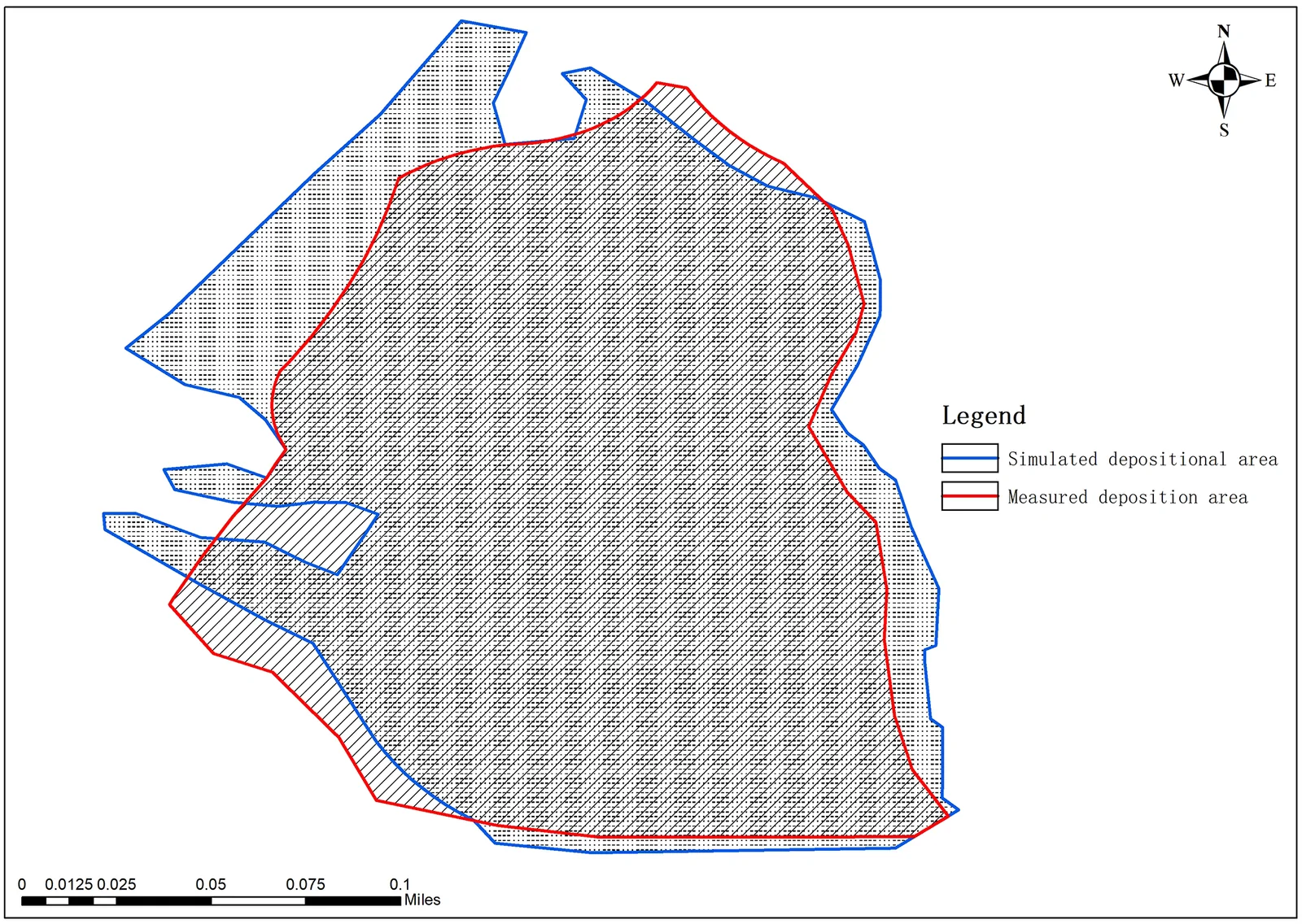
Numerical simulation validation for the 2024 scenario (deposition area).

The measured accumulation area and mud trace height are used to validate the 2024-condition debris flow model in this study [45]. A comparative analysis between the simulated maximum depth (2.73m) and field measurements (2.43m) yields a simulation accuracy of 89.01%. The numerically simulated area of the deposition zone is 0.059 km². Field reconnaissance and site investigations reveal that the measured deposition area of the 2024 debris flow event is 0.051 km². Superposition comparison between the simulated and measured extents (Fig 11) indicates a simulation accuracy of 87.84%. These results demonstrate that the FLO-2D model effectively replicates the kinematic laws of debris flows in steep-gully environments, confirming the high reliability of the simulation framework.

#### 4.4.2. Simulation analysis of debris flow deposition depth.

**Fig 12:**
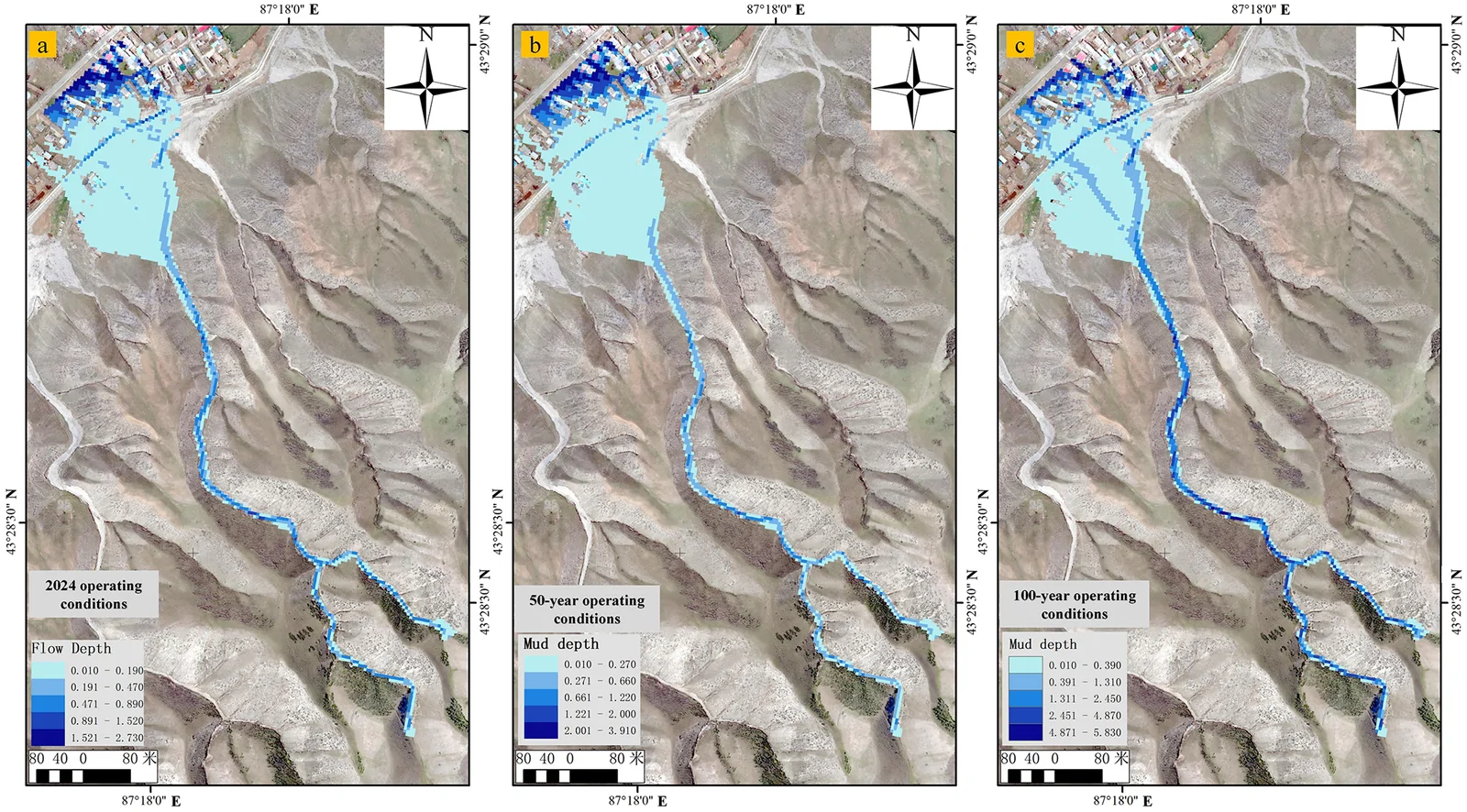
Distribution of debris flow depth under three working conditions (a 2024 working condition; b 2% rainfall event; c 1% rainfall event). (The base map was generated by the authors using field UAV survey data).

Numerical simulations using FLO-2D provided flow depth results for three scenarios: the 2024 historical event, a 50-year return period (P = 2%), and a 100-year return period (P = 1%). The results (Fig 12) indicate that the maximum flow depth for the 2024 scenario was 2.84 m, while the maximum depths for the 50-year and 100-year events reached 3.91 m and 5.83 m, respectively.

1. Longitudinal Distribution Characteristics

In the formation and transportation zones, the flow was characterized by rapid material transport with generally shallow depths, owing to the narrow, straight channels and high longitudinal gradient (average 266.3‰). Under the 2024, 50-year, and 100-year scenarios, the average flow depths in these sections were 0.62 m, 0.89 m, and 1.35 m, respectively, exhibiting minimal variation along the channel. This suggests that the steep gully geometry exerts a strong constraining effect, concentrating energy on gravitational-to-kinetic energy conversion rather than deposition.

2. Deposition Patterns in the Accumulation Area

As the debris flow transitions from the “Y”-shaped gully into the trumpet-shaped alluvial fan at the gully mouth, the flow velocity drops sharply, triggering the rapid sedimentation of entrained solid materials. The high-depth clusters are primarily concentrated north of the main canal in the front-middle section of the fan, directly threatening residential settlements.

2024 Scenario: The average depth in the accumulation area reached 1.87 m, a significant increase relative to the upstream sections, with a peak of 2.84 m recorded at residences adjacent to the main canal (Fig 12a).

50-year Scenario: The average depth rose to 2.73 m. The peak-depth zone extended northward by approximately 35 m along the fan’s central axis, with the maximum depth of 3.91 m localized in a depression 12 m northwest of the original peak (Fig 12b).

100-year Scenario: The average depth further increased to 4.15 m. The high-depth zones expanded remarkably, stretching 82 m northward and extending 45 m and 58 m laterally (east and west). The maximum depth of 5.83 m occurred at a residential structure in the north-central part of the fan (Fig 12c).

A planimetric comparison reveals that as the rainfall return period increases, both the inundation area and the deposition thickness exhibit a significant upward trend. The expansion of high-depth zones aligns with the topographic slope, progressing northward and northwestward along the gentle central axis—a pattern consistent with energy diffusion and material sorting laws governed by trumpet-shaped terrains.

3. Vertical Variation and Amplification Effects

Vertical analysis at identical locations reveals a nonlinear amplification effect in deposition thickness under extreme rainfall. The depth for the 100-year event is approximately 1.7 times that of the 50-year event, while the 50-year depth is 1.47 times that of the 2024 scenario. This phenomenon is driven by the synergistic increase in rainfall intensity, peak discharge, and material mobilization, which significantly boosts the total sediment volume reaching the fan. Furthermore, the spatial attenuation of hydraulic energy during diffusion results in a distribution pattern where material carrying capacity—and thus deposition thickness—decreases from the center toward the periphery.

#### 4.4.3. Simulation analysis of debris flow velocity.

**Fig 13:**
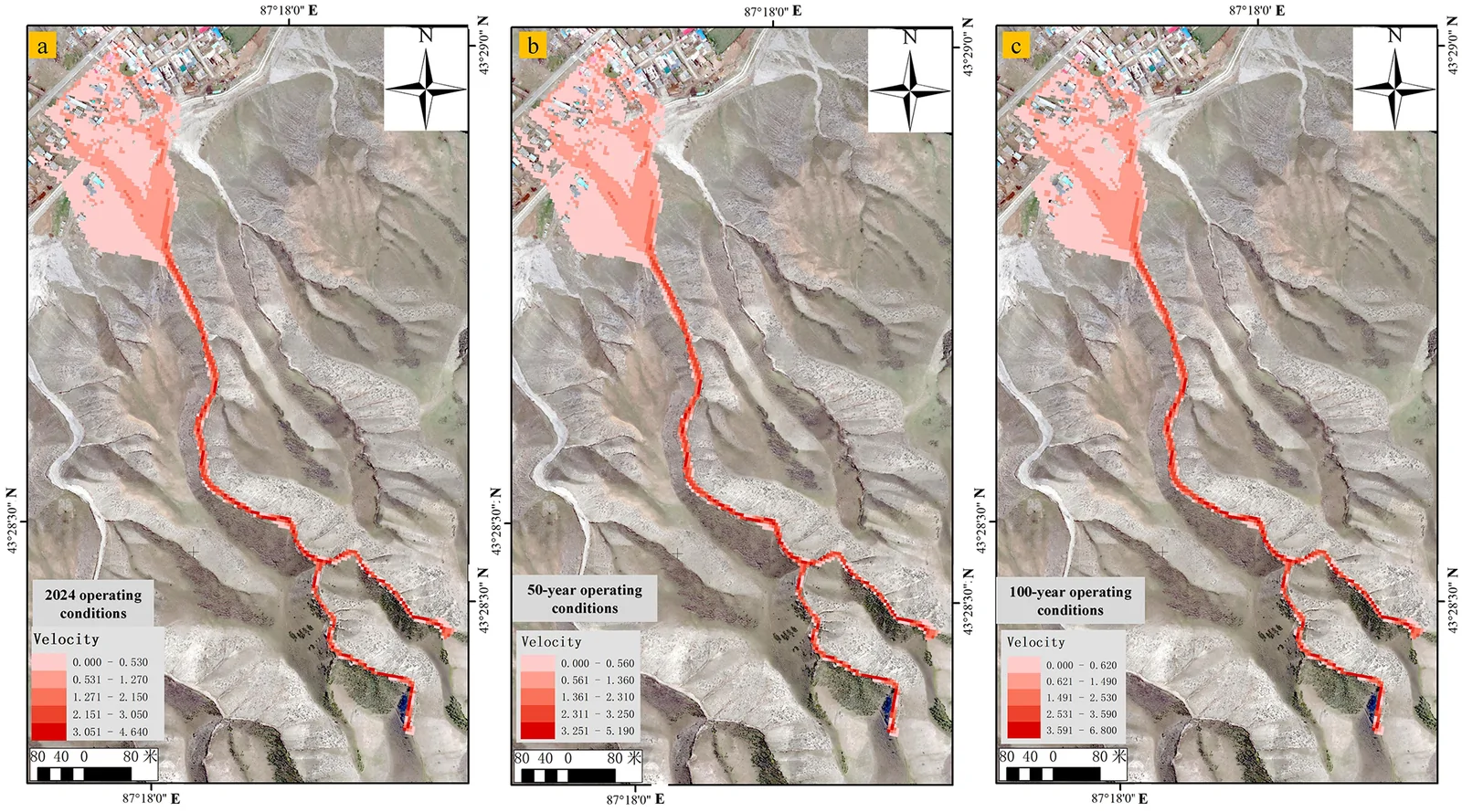
Distribution of debris flow velocity under three working conditions (a 2024 working condition; b 2% rainfall event; c 1% rainfall event). (The base map was generated by the authors using field UAV survey data).

Numerical simulation results from FLO-2D indicate a positive correlation between rainfall intensity and debris flow velocity. As illustrated in Fig 13, under the 2024 scenario, the average velocity in the formation and transportation zones was approximately 3.63 m/s, dropping sharply to 2.15 m/s upon entering the accumulation area. The peak velocity of 4.79 m/s was localized in the narrow gully section of the middle formation zone (Fig 13a).

Under the 50-year return period, the average velocity in the formation and transportation zones increased to 4.28 m/s, while the velocity in the accumulation area rose to 2.76 m/s, with a maximum velocity of 5.19 m/s at the transition between the formation and transportation zones (Fig 13b). For the 100-year return period, the average velocity in the upstream sections climbed to 5.25 m/s, the accumulation area velocity reached 3.32 m/s, and a maximum velocity of 6.8 m/s was recorded at the steep slope of the initial formation section (Fig 13c).

1. Spatial Velocity Distribution and Morphological Control

Across all three scenarios, a consistent spatial pattern emerged: high velocity in the formation and transportation zones and low velocity in the accumulation area. This distribution highlights the lateral constraint provided by the narrow and straight gully channels, which minimizes friction and facilitates high-velocity transport. Conversely, the sharp attenuation of velocity in the accumulation area is attributed to momentum dissipation caused by the sudden expansion of the cross-sectional area as the flow exits the gully mouth.

2. Velocity Growth Rates and Rainfall Sensitivity

Comparative analysis of velocity growth rates reveals distinct sensitivities to rainfall intensity. Transitioning from the 2024 scenario to the 50-year event, the average velocity in the formation/transportation zones and the accumulation area increased by 17.9% and 28.4%, respectively. From the 50-year to the 100-year event, the increases were 22.7% and 20.3%, respectively. These data suggest that as rainfall intensity escalates, the growth rate of velocity becomes more pronounced in the formation and transportation zones. This underscores the stimulating effect of extreme rainfall on flow dynamics within narrow, steep gully environments.

3. Coupling of Velocity and Deposition Depth

Superimposing the velocity simulation results with the flow depth distribution reveals a clear inverse correlation: areas of high velocity correspond to minimal deposition depth, whereas low-velocity zones correlate with significant accumulation. This spatial coupling validates the kinematic mechanism characteristic of steep-gully debris flows: high-velocity transport in the upstream reaches and low-velocity sedimentation upon reaching the open terrain of the alluvial fan.

#### 4.4.4. Hazard assessment.

The velocity and flow depth of a debris flow are the primary determinants of its destructive intensity. Hazard analysis serves as a critical hazard management tool for identifying vulnerable areas and optimizing mitigation strategies. This study integrates the hazard zonation methodologies proposed by Yang et al. [46] and Wu et al. [47], which were specifically developed based on debris flow dynamics in steep, high-gradient gully channels. These methods account for unique kinematic features such as high flow velocities, rapid discharge concentration, and significant longitudinal variations in flow depth.A threshold system integrating flow velocity (Vc) and flow depth (Hc) was adopted (Table 1), which has demonstrated high adaptability in practical engineering applications. Based on these thresholds, the hazard extent under different rainfall frequencies was demarcated, and the hazard intensity for the three scenarios was classified into distinct levels (Fig 13). Utilizing the ArcGIS platform, a spatial overlay analysis was performed to calculate the total area and the corresponding proportions of each hazard level (Table 2).

**Table 1:** Hazard Zoning Criteria for Debris Flow.

Hazard	Accumulation mud depth (m)	Logical relation-ship	Product of the mud depth accumulation and velocity
High	*H*_*c*_≥ 1.5	OR	*V*_*C*_*H*_*c*_ ≥ 1.5
Medium	0.5 < *H*_*c*_ < 1.5	AND	0.5 < *V*_*C*_*H*_*c*_ < 1.5
Low	0.01 ≤ *H*_*c*_ ≤ 0.5	AND	0.1 ≤ *V*_*C*_*H*_*c*_ ≤ 0.5

**Table 2:** Statistics of numerical simulation results and hazard assessment areas across three scenarios.

Snowmelt conditions/Rainfall frequency (P)	Low		Medium		High	
	Area(10^4^m^2^)	Ratio	Area(10^4^m^2^)	Ratio	Area(10^4^m^2^)	Ratio
2024a	3.48	74.68%	0.56	12.02%	0.62	13.30%
2%	3.65	71.57%	0.6	11.76%	0.85	16.67%
1%	3.13	49.21%	0.95	14.94%	2.28	35.85%

**Fig 14:**
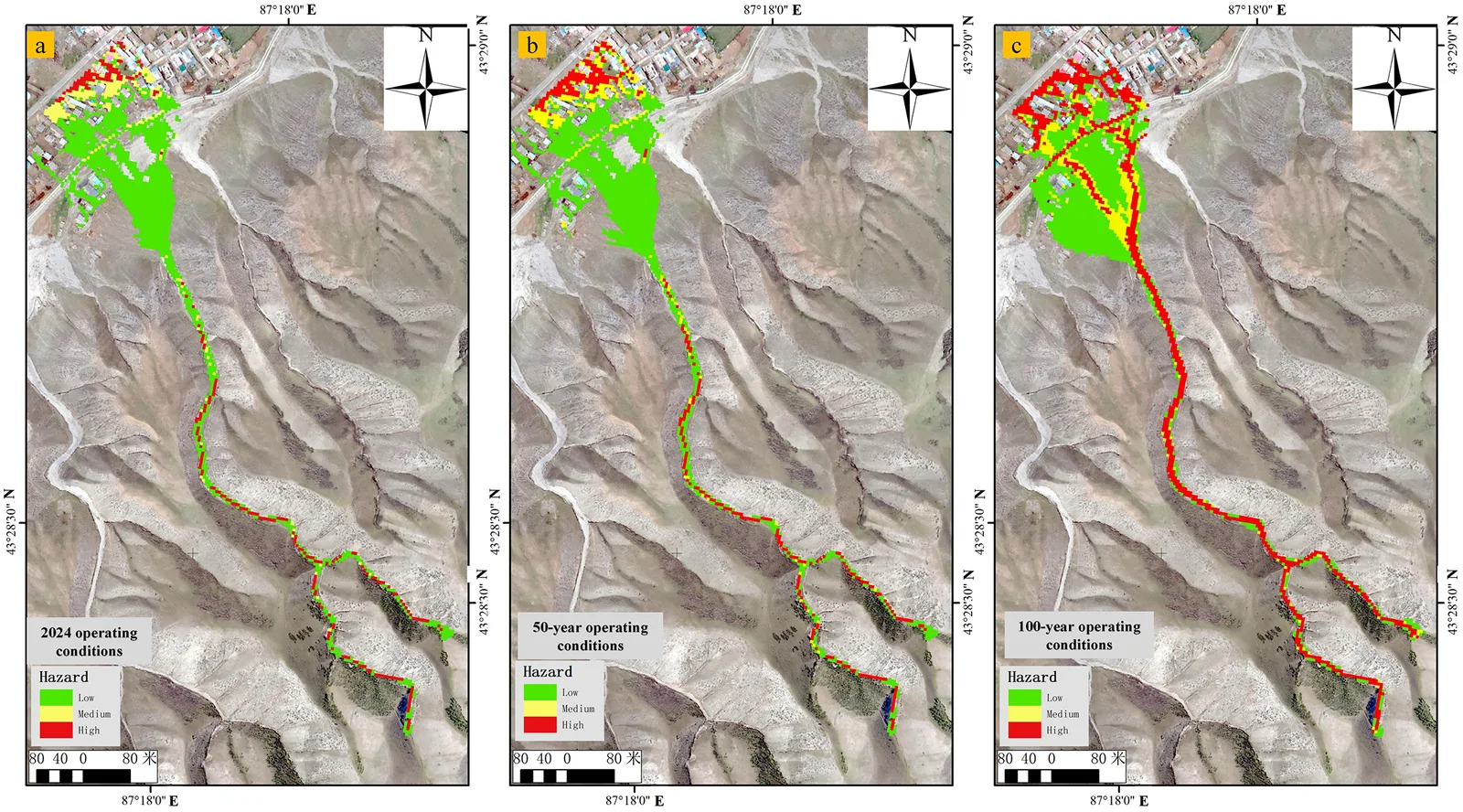
Distribution of debris flow hazard under three working conditions (a 2024 working condition; b 2% rainfall event; c 1% rainfall event). (The base map was generated by the authors using field UAV survey data).

According to the hazard zonation results (Fig 14), under the 2024 scenario, the high-hazard zone was primarily concentrated in the core residential area north of the main canal within the front-central part of the accumulation fan. This zone covered an area of 0.62 × 10^4^m^2^, accounting for 13.30% of the total affected area. This distribution is highly consistent with the actual locations of severely damaged structures during the 2024 event, validating the reliability of the zonation criteria. The medium-hazard zone occupied a smaller area of 0.56 × 10^4^m^2^ (12.02%), mainly distributed as a transition belt around the periphery of the high-hazard zone. In this area, flow depths ranged from 0.5 to 1.5m, posing a moderate threat to low-rise structures and infrastructure. The low-hazard zone was the most extensive, covering 3.48 × 10^4^m^2^ (74.68%), and was widely distributed across the formation, transportation, and fan-edge areas, characterized primarily by minor sedimentation or splash erosion with limited destructive potential.

Under the 50-year return period (P = 2%), the area of the high-hazard zone increased to 0.85 × 10^4^m^2^(16.67%). Compared to the 2024 scenario, its extent expanded approximately 35m northward along the fan’s central axis and slightly westward, mirroring the expansion of high-depth clusters under this scenario. This indicates that increased rainfall intensity significantly broadens the direct threat to residential areas. The medium-hazard zone covered 0.6 × 10^4^m^2^ (11.76%), maintaining a similar spatial pattern but with a slight areal increase. This growth resulted from the elevation of former low-hazard areas to medium-hazard status due to increased velocity and depth. The low-hazard zone, while still dominant at 3.65 × 10^4^m^2^(71.57%), saw a decrease in its relative proportion.

Under the 100-year return period (P = 1%), the hazard intensity exhibited a dramatic shift. The high-hazard zone surged to 2.28 × 10^4^m^2^, accounting for a substantial 35.85% of the total affected area. Its scope extended 82m northward and reached 45m eastward and 58m westward, encompassing nearly the entire residential area and adjacent cultivated land. The residential structure where the maximum flow depth reached 5.83m was located at the core of this zone. The medium-hazard zone expanded to 0.95 × 10^4^m^2^ (14.94%), forming a broad transition belt reflecting the gradual attenuation of energy during diffusion. Conversely, the low-hazard zone was drastically reduced to 3.13 × 10^4^m^2^, with its proportion dropping to 49.21%, localized mainly in the upstream reaches and the outermost fan edges.

A comprehensive analysis reveals that with increasing return periods, the proportions of both high and medium-hazard zones exhibit an upward trend. The high-hazard zone shows the most remarkable growth, rising from 13.30% in the 2024 scenario to 35.85% in the 100-year event—an increase of 22.55 percentage points. In contrast, the low-hazard zone’s proportion declined from 74.68% to 49.21%. This evolutionary pattern indicates that extreme rainfall events significantly exacerbate debris flow hazard in steep-gully systems, posing a severe threat particularly to residential clusters on the alluvial fan.

## 5. Discussion

This study utilized the FLO-2D model to simulate debris flow dynamics in high-gradient, steep gully channels, revealing distinct kinematic characteristics and hazard patterns across various rainfall return periods. The results underscore that topographic modulation is the primary driver of debris flow evolution. In the formation and transportation zones, the narrow, straight channel morphology combined with high longitudinal gradients (averaging 266.3‰) facilitates efficient kinetic energy maintenance, resulting in high flow velocities and minimal sedimentation. Conversely, upon entering the accumulation area, the abrupt transition from a constrained “Y”-shaped channel to an expansive, trumpet-shaped alluvial fan triggers a sharp drop in velocity. This leads to a rapid kinetic-to-potential energy conversion and the massive deposition of solid materials, which explains why mud marks observed in the accumulation zone are significantly thicker than those in upstream sections.

The selection and calibration of simulation parameters are pivotal for model fidelity. This study determined that the combination of volume concentration (Cv), laminar flow resistance (K), and Manning’s roughness (n) effectively captures the physio-mechanical properties of the debris flow. Sensitivity analysis indicates that an increase in Cv tends to elevate the simulated flow depth while reducing velocity due to increased internal friction. Furthermore, K directly governs rheological accuracy, where excessive values may lead to an underestimation of flow velocity.

Given that steep-gully debris flows often exhibit high velocities and solid concentrations, their behavior may deviate from an ideal Bingham [48] fluid. Future research should introduce more complex rheological models, such as the Herschel-Bulkley [49] model, to better capture these non-Newtonian nuances. Additionally, accounting for the spatial heterogeneity of Manning’s n across different segments (e.g., bedrock vs. loose sediment) remains a priority for refining velocity simulations.

The study reveals a composite disaster-causing mechanism characterized by “upstream dynamic control and downstream topographic restriction.” While the upstream regions provide the material basis and kinetic energy, the downstream trumpet-shaped terrain acts as a bottleneck for energy dissipation and material siltation, directly impacting residential clusters. Comparative analysis across return periods demonstrates a nonlinear amplification effect: as rainfall intensity increases, peak discharge and deposition depth grow disproportionately. This is particularly evident in the accumulation zone, where high-depth clusters expand exponentially, embodying the heightened threat posed by extreme rainfall events.

The hazard zonation results align closely with high-value areas of flow depth and velocity. As the return period increases, high-hazard zones not only expand in area but also migrate northward and northwestward along the gentle central axis of the alluvial fan, following the topographic slope. While the current assessment relies on physical parameters (the product of h × v), future iterations could incorporate building vulnerability to transition from a physical hazard model to a more integrated socio-economic hazard assessment.

To translate these dynamic simulations into practical disaster hazard reduction strategies, the spatial layout of mitigation structures must be prioritized. According to the FLO-2D outputs, the topographic “choke points” in the middle-upper reaches, where flow velocity sharply accelerates, should be prioritized for installing flexible ring-net barriers to dissipate kinetic energy. Conversely, the transition zone at the gully outlet, where the flow depth reaches its maximum peak, must be the primary focus for constructing rigid check dams and diversion embankments to safely channel the runout volume away from downstream settlements.

While the FLO-2D model successfully delineated the hazard zones and captured the macro-kinematic characteristics of the debris flow, it remains fundamentally a fixed-bed hydrodynamic model. Actual debris flow propagation is frequently accompanied by intense dynamic mass entrainment, gully-bed scouring, and continuous topographic modifications. The current simulation framework does not account for these transient riverbed adjustments and continuous material supply, which may lead to conservative depth estimations in severe erosion zones and an underestimation of the overall disaster scale during extreme events. To address these limitations, future research should integrate fully coupled morphodynamic models that explicitly consider bed erosion, sediment yield, and real-time topographic evolution. Furthermore, incorporating multi-hazard coupling mechanisms—such as the triggering effects of glacial meltwater and seismic activity—will be essential to provide a more comprehensive regional disaster hazard assessment.

## 6. Conclusions

By integrating field geological surveys, laboratory testing, and FLO-2D numerical modeling, this study systematically characterized the formative conditions, movement dynamics, and hazard patterns of debris flows in the high-gradient, steep gully channels of the study area. The primary conclusions are summarized as follows:

(1) The initiation of debris flows in these steep gully systems is synergistically controlled by the geo-environmental framework and specific rainfall thresholds. Structurally fractured rock masses and abundant loose soil provide the requisite material base, with colluvial, proluvial, and gully deposits serving as the primary sources. The basin’s topographic profile—characterized by narrow, straight channels and high longitudinal gradients—provides optimal conditions for rapid discharge concentration and long-distance transport. For the 2024 event, a total daily rainfall of 90.2m and a peak hourly intensity of 63.2 mm acted as the decisive meteorological triggers.
(2) The disaster-causing mode in these high-gradient channels is a coupled result of topography, lithology, and hydro-meteorology. This study elucidates a mechanistic sequence of “source enrichment→dynamic triggering→channel transmission→deposition-induced impact.” Loose materials in the formation area are mobilized by heavy rainfall, accelerated through steep transportation channels to form high-velocity flows, and finally undergo rapid energy dissipation and mass siltation upon entering the open downstream alluvial fan, directly impacting residential clusters.
(3) Utilizing the 2024 historical event as a benchmark, the FLO-2D model was rigorously validated through a dual-metric approach. The comparative analysis yielded an accuracy of 89.01% for the maximum flow depth (kinematic) and an 87.84% spatial overlap for the deposition area (morphological). This high degree of consistency between the simulation and actual observations confirms the reliability and effectiveness of the proposed modeling framework for rapid and steep-gully systems.
(4) Numerical results from FLO-2D indicate that debris flow intensity and hazard extent escalate significantly with increasing rainfall return periods. For the 100-year scenario, the maximum flow depth reaches 5.83m (up 51.3% from 2024), with high-depth zones expanding markedly along the fan’s central axis. The peak velocity climbs to 6.80m (up 29.6% from 2024), maintaining a steady upward trend even in the accumulation area. Consequently, the high-hazard zone nearly triples in area, surging from 13.30% to 35.85%, which poses a critical threat to residential settlements in the study area.
